# Role of Gist and PHOG Features in Computer-Aided Diagnosis of Tuberculosis without Segmentation

**DOI:** 10.1371/journal.pone.0112980

**Published:** 2014-11-12

**Authors:** Arun Chauhan, Devesh Chauhan, Chittaranjan Rout

**Affiliations:** 1 Department of Biotechnology and Bioinformatics, Jaypee University of Information Technology, Solan, Himachal Pradesh, India; 2 Department of Radiology, National Institute of TB and Respiratory Diseases, New Delhi, India; University of Ulm, Germany

## Abstract

**Purpose:**

Effective diagnosis of tuberculosis (TB) relies on accurate interpretation of radiological patterns found in a chest radiograph (CXR). Lack of skilled radiologists and other resources, especially in developing countries, hinders its efficient diagnosis. Computer-aided diagnosis (CAD) methods provide second opinion to the radiologists for their findings and thereby assist in better diagnosis of cancer and other diseases including TB. However, existing CAD methods for TB are based on the extraction of textural features from manually or semi-automatically segmented CXRs. These methods are prone to errors and cannot be implemented in X-ray machines for automated classification.

**Methods:**

Gabor, Gist, histogram of oriented gradients (HOG), and pyramid histogram of oriented gradients (PHOG) features extracted from the whole image can be implemented into existing X-ray machines to discriminate between TB and non-TB CXRs in an automated manner. Localized features were extracted for the above methods using various parameters, such as frequency range, blocks and region of interest. The performance of these features was evaluated against textural features. Two digital CXR image datasets (8-bit DA and 14-bit DB) were used for evaluating the performance of these features.

**Results:**

Gist (accuracy 94.2% for DA, 86.0% for DB) and PHOG (accuracy 92.3% for DA, 92.0% for DB) features provided better results for both the datasets. These features were implemented to develop a MATLAB toolbox, TB-Xpredict, which is freely available for academic use at http://sourceforge.net/projects/tbxpredict/. This toolbox provides both automated training and prediction modules and does not require expertise in image processing for operation.

**Conclusion:**

Since the features used in TB-Xpredict do not require segmentation, the toolbox can easily be implemented in X-ray machines. This toolbox can effectively be used for the mass screening of TB in high-burden areas with improved efficiency.

## Introduction

X-rays were discovered by Wilhelm Röntgen, a German physicist in 1895 and have revolutionized the field of diagnostics. Chest radiographs (CXRs) are ubiquitous in clinical diagnostics and make up about one-third of all radiological examinations. Chest radiographs are inexpensive and least invasive primary diagnostic tool for tuberculosis (TB). They are used in routine medical checkups and immigrant medical examinations even in well-equipped hospitals where blood and skin tests are available [1]. The CXRs are also used for mass screening of TB in human immunodeficiency virus (HIV) endemic areas.

Tuberculosis is one of the leading infectious diseases with high mortality rate in developing and under-developed countries. Approximately 1.1 and 0.35 million of deaths were caused worldwide by the disease in non-HIV and HIV people respectively from about 8.8 million incidents reported in 2011 [2]. The disease is diagnosed on the basis of patient's symptoms, CXR and smear microscopy tests. Since the accuracy of smear microscopy test has been less than 50%, diagnosis relies primarily on the interpretation of radiological patterns found in a CXR. Early detection of TB often leads to success of anti-TB therapy, and also helps in gaining control over the transmission of infection as well as development of drug resistant TB. However, lack of resources and skilled radiologists particularly in rural areas of developing countries impedes its effective diagnosis. Therefore, computer-aided diagnosis (CAD) tools assume a lot of significance as they not only reduce diagnostic errors but also increase the efficiency of mass screening in poor-resource settings.

The CAD may be referred to a diagnosis made by a radiologist taking into account the results obtained through computer analysis. Success of the CAD methods in better diagnosis of breast cancer, colon cancer, lungs cancer, prostate cancer, coronary artery disease, congenital heart defects, etc., has suggested that TB may be detected more effectively by incorporating CAD methods [3]–[6]. The CAD techniques have been helpful in providing second opinion to the radiologists for their findings as well as assisting them in making their final decision regarding diagnosis with increased accuracy. Interpretation of CXRs is dependent on the expertise and skills of the radiologist, which is subject to human error. Even many district level hospitals in developing countries do not have skilled radiologists and incorrect diagnosis of TB takes place due to erroneous interpretation of CXR patterns. A well-trained CAD method can assume the role of a second CXR reader to some extent and may assist the radiologist in some facets of disease detection and decision making. Although these techniques may by no means be able to achieve the level of cognitive ability and knowledge of a radiologist, nonetheless, a trained classifier can perform the prediction consistently without the intra-observer and inter-observer variability. A recent study has also shown that the performance of CXR readers in diagnosing TB improved with the support of CAD [7].

Basic methodology employed in most of the earlier reported CAD studies was segmentation and extraction of grey-level co-occurrence matrix (GLCM) textural features [8]. Ginneken *et al*. used the GLCM textural features on two datasets for classification of CXRs as TB or non-TB, but the performance of both varied significantly [9]. For one dataset, specificity of 90% was obtained while only 50% was achieved for the other. Detection of clavicle, and abnormalities in texture and shape in the CXRs were combined to develop a TB classification model by Hogeweg *et al* [10]. The area under the receiver operating characteristic curve (AUC) for the method was 0.86 [9]. Semi-automated segmentation based classification model using textural features provided a prediction accuracy of 92.9% with sensitivity of 0.91 [1].

**Figure 1:**
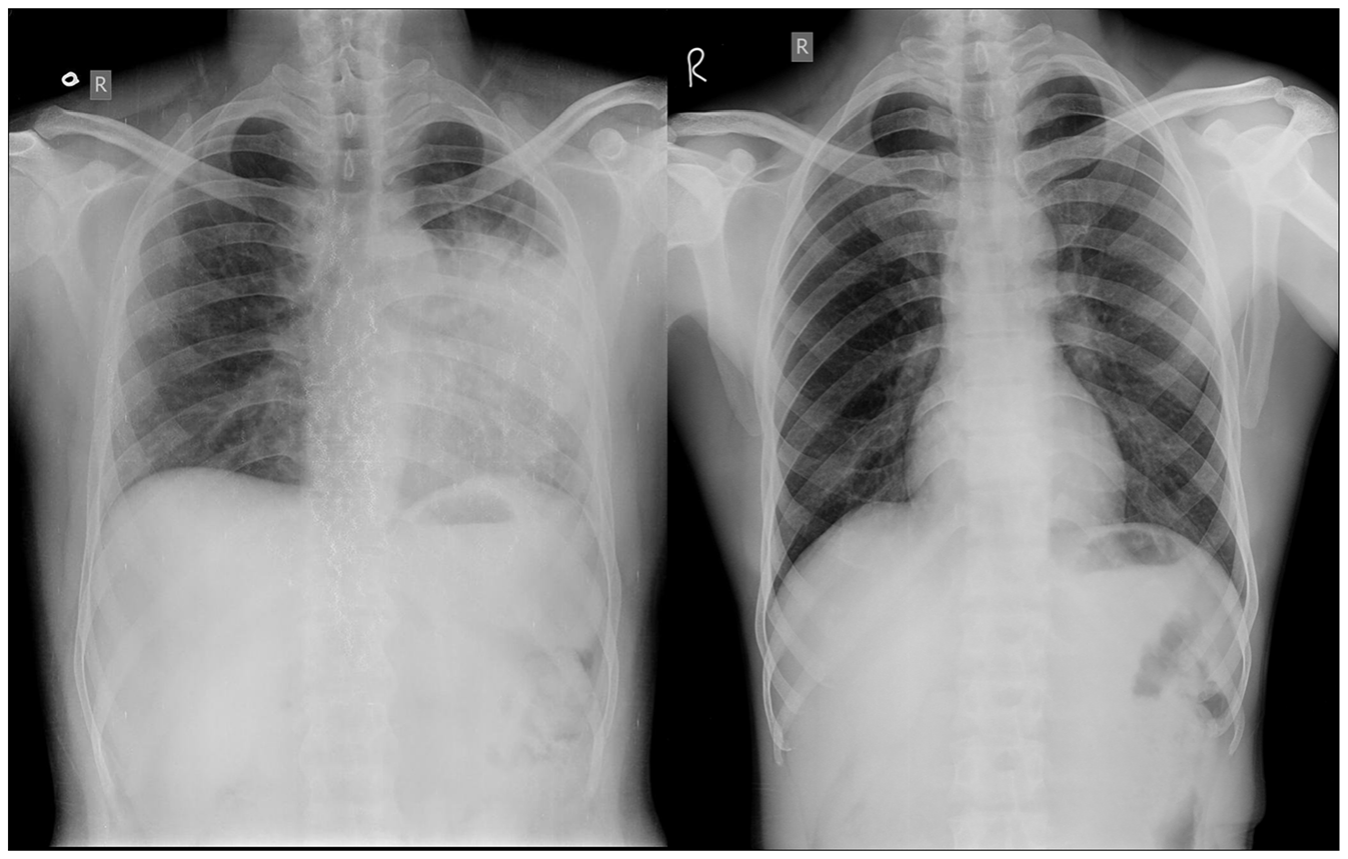
CXRs. Left: TB patient Right: Normal person.

Current CAD methods employed for classification of CXR images are either object or region based [11]–[13]. These methods require segmentation and are prone to magnifying and carrying over low-level errors. The performance of GLCM features was inconsistent across various datasets [9], and these features lose significance when lungs have different shapes or CXRs have complex appearances due to overlapping of anatomical structures [1]. Commonly used manual or semi-automatic segmentation methods not only require high expertise but also make the classification model data and machine dependent. Although automated segmentation methods have potential to be used for development of CAD techniques, but studies have shown that automated segmentation of TB CXRs failed in many cases leading to incorrect classification [1]. Textural features are based on the implicit hypothesis that there exist some specific texture signatures which are dissimilar between non-TB and TB CXRs (Figure 1). The effectiveness of these textural signatures greatly depends on their ability to correlate with the disease. Since context of the features obtained with respect to the whole image are expected to be more useful than local textural features, therefore, contextual scene based Gist features were used in this study for the prediction of TB from CXR. This method captures biologically plausible features into a signatory low-dimensional vector [14]. These features identify salient locations within the image which differ significantly from their neighbours and also accumulate image statistics over the entire scene. Gabor [15], histogram of oriented gradients (HOG) [16], and pyramid histogram of oriented gradients (PHOG) [17] features were also used in this study. PHOG features were computed as they contain local as well as global spatial information. The advantage with these methods is that they can be incorporated to X-ray machines for fully automated detection of TB. Localisation of features was done through optimising and fine-tuning various parameters, like region of interest (ROI), blocks and frequency range.

## Materials and Methods

**Figure 2:**
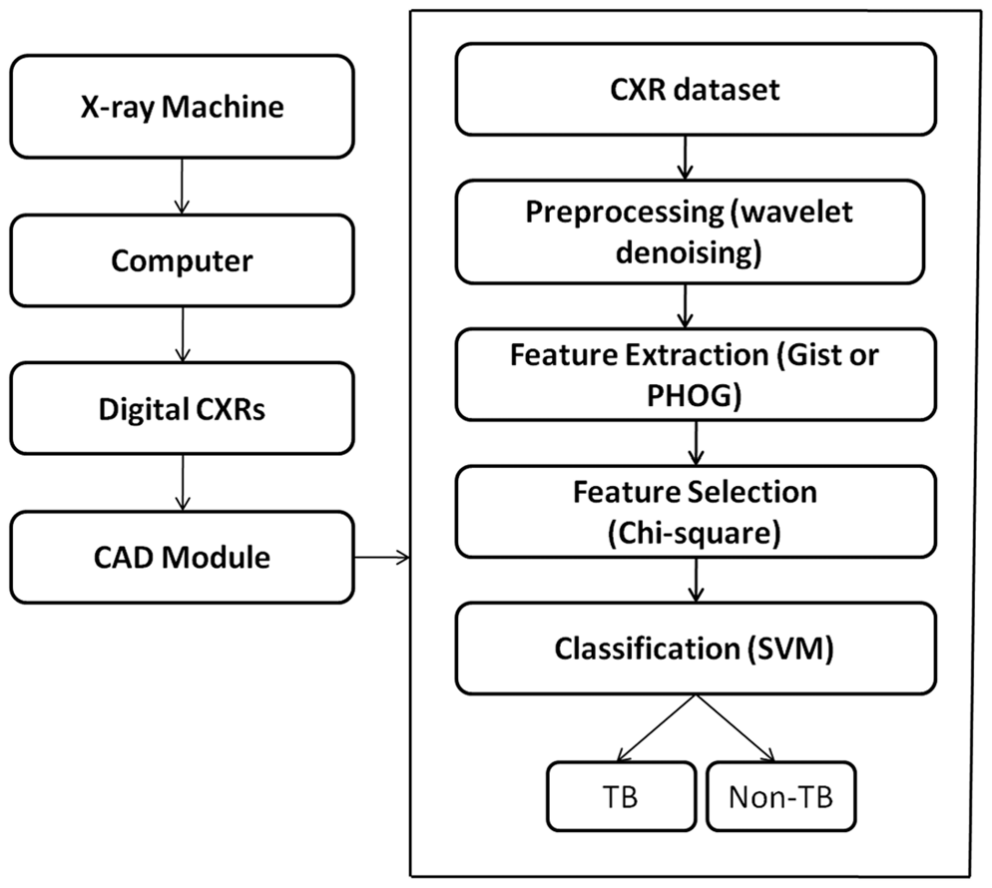
Schematic representation of CAD implementation for TB. CAD module is applied to the digital CXRs obtained from the computer attached to the the X-ray machine. The CAD module preprocesses (denoises) the image using SURE wavelet denoising, extract features (Gist and PHOG), selects relevant features using chi-square based feature selection method and finally classifies the CXR into TB and non-TB.

The methodology is based on pixel-based classification which is illustrated in Figure 2. The proposed methodology was implemented in MATLAB 2010a.

### Ethics statement

This study was approved by the institution, National Institute of TB and Respiratory Diseases, and the requirement to obtain informed consent was waived. The data was obtained and analysed anonymously.

### Dataset

Two CXR digital image datasets were obtained from two different X-ray machines available at the National Institute of Tuberculosis and Respiratory Diseases, New Delhi. These CXRs were taken in standard practice conditions followed by clinicians. In developing countries like India, the posterior-anterior (PA) CXRs are mostly taken and lateral CXRs are taken rarely. Therefore, only PA CXRs were used in the creation of the datasets. The CXRs were randomly collected over a period of one year with varied lung presentations. Dataset A (DA) was taken from Diagnox-4050 X-ray machine manufactured by Meditronics with CXRs digitised by AGFA CR35-X. Dataset B (DB) was obtained from PRORAD URS with Canon CXDI detectors. The tube voltage was set to 50 kV and 55 kV for DA and DB respectively. E-7252X and E-7254X X-ray tubes manufactured by Toshiba, Japan were used by Diagnox-4050 and PRORAD URS respectively. Although AGFA CR35-X acquires 12-bit grayscale CXRs, but 8-bit CXRs (DA) were obtained due to imaging system used by the radiologists. 14-bit CXR digital images (DB) were obtained by using PRORAD URS. For both the datasets DA and DB, the acquired images were resized to 1024×1024 resolutions to obtain images with identical dimensions. No change in classification accuracy was noticed with respect to original sized images. CXRs from each dataset were randomly divided into training and test sets. DA comprised of training set (52 non-TB and 52 TB CXRs) and the independent test set (26 non-TB and 26 TB CXRs). Similarly, DB comprised of training set (50 non-TB and 50 TB CXRs) and the independent test set (25 non-TB and 25 TB CXRs). Selection of all non-TB and TB cases was based on the consensus of independent review of each CXR by two highly experienced chest radiologists from National Institute of Tuberculosis and Respiratory Diseases, New Delhi, India and Indira Gandhi Medical College, Shimla, India. The selection of abnormal CXRs, varying from subtle to severe TB findings, was based on radiological findings through the consensus of the radiologists. Datasets are available freely at http://sourceforge.net/projects/tbxpredict/files/data/.

### Segmentation

**Figure 3:**
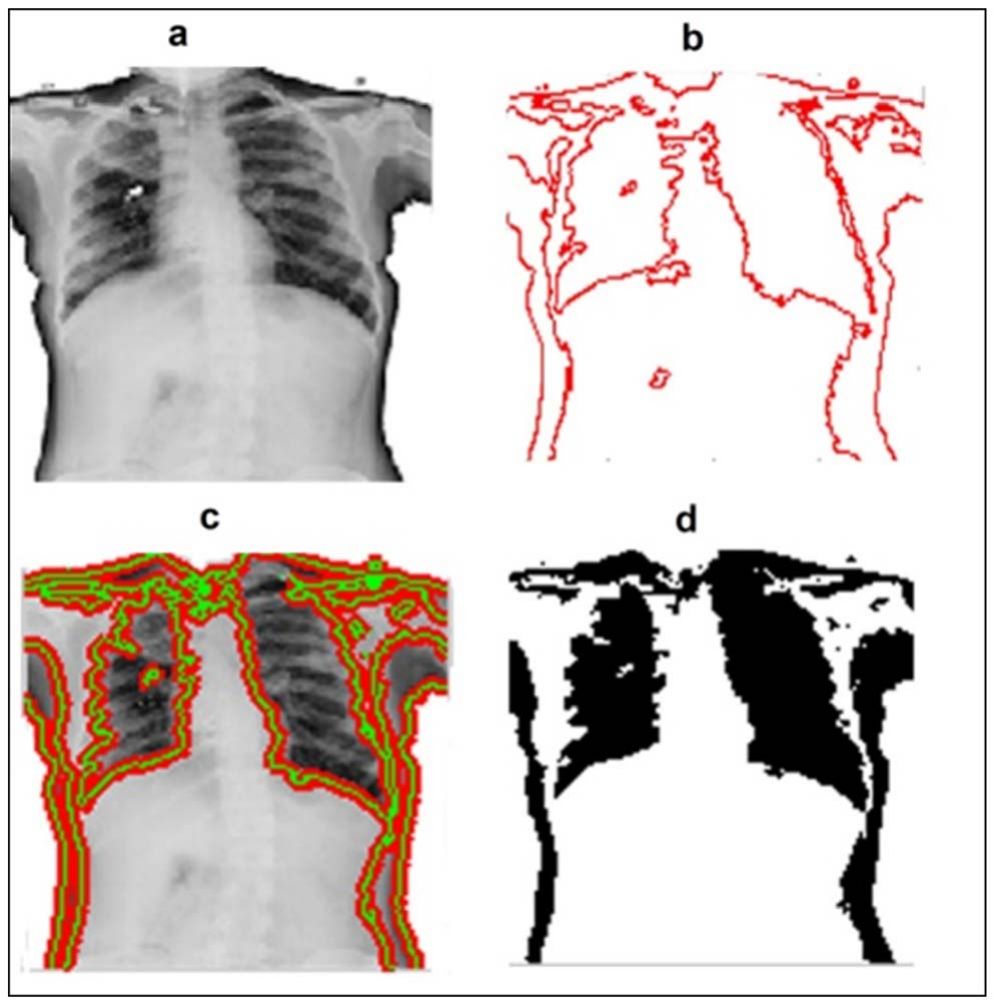
Chan-Vese active contour segmentation of a CXR. a. Original CXR image b. Initial contour c. Iterations for foreground segmentation, and d. Segmented image.

Segmentation is the process of dividing an image into a set of distinctive areas or regions that differ significantly qualitatively or quantitatively. It is a critical intermediate step in high level object-recognition tasks. Since existing methods required segmentation of CXRs, manual and automatic segmentation of CXRs for the dataset DA was done merely to evaluate the performance of our method (features extracted from whole image) against features extracted from manual and automatic segmentation. Manual segmentation of CXRs was done with the help of ImageJ software [18]. To evaluate the effectiveness of classification using features extracted from automated segmentation methods, Chan-Vese method [19] was used for the segmentation of CXRs from the dataset DA (Figure 3). The Chan-Vese algorithm is a type of geometric active contour model which begins with a contour in the image plane defining an initial segmentation, and then this contour evolves with respect to evolution equation. The contour is evolved using level set method in such a manner that it terminates on the foreground boundaries. The level set function minimizes the Mumford-Shah functional, which is a type of “fitting energy” functional.

**Figure 4:**
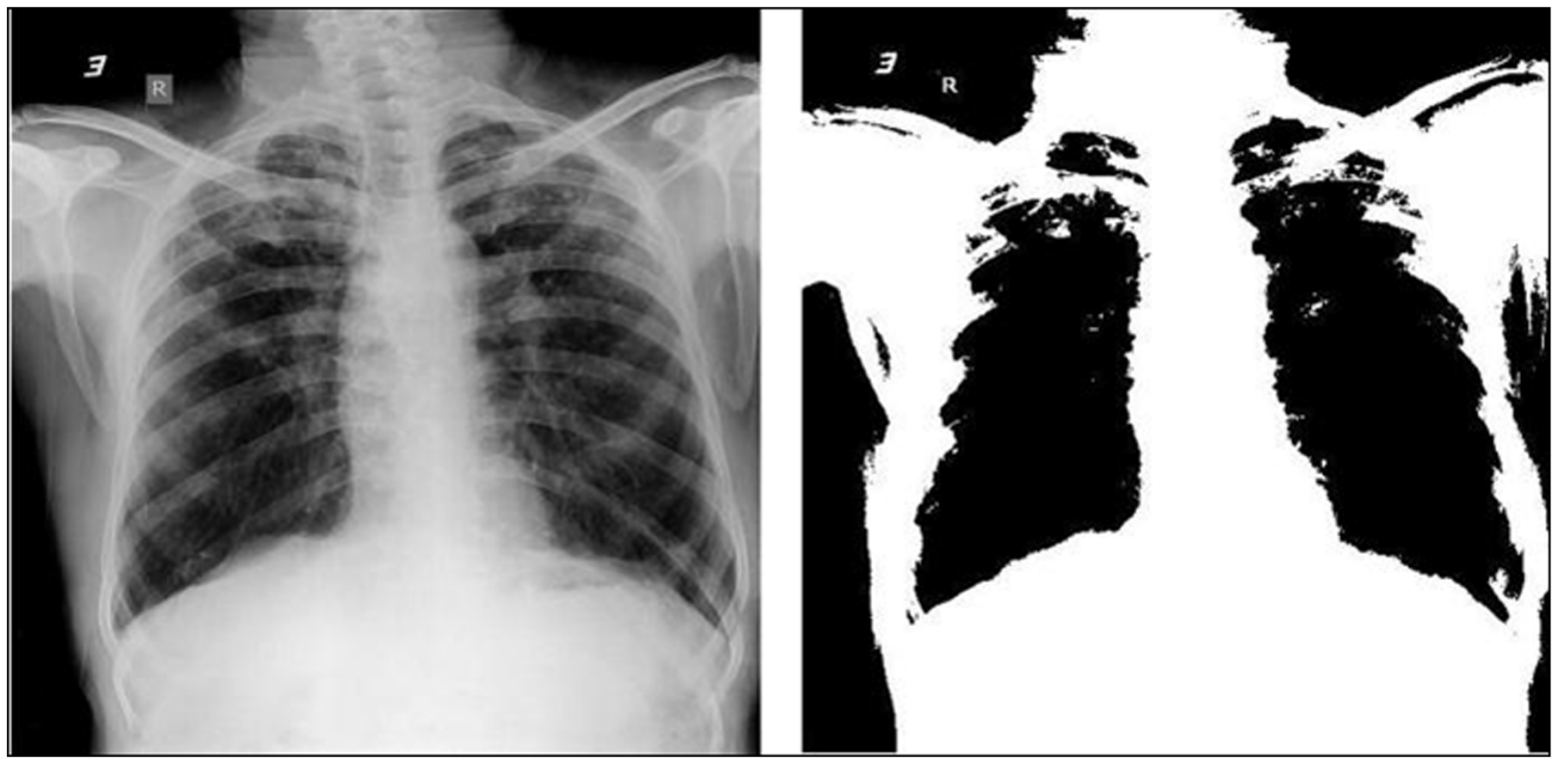
X-ray image before and after Euler-based segmentation. The Euler-based segmented image provides initial contour for Chan-Vese segmentation.

Initial contour was developed using Euler-number [20] based segmentation with an auto-tuned threshold value as shown in Figure 4. The optimal threshold value for this segmentation is obtained on the basis of minimum and maximum values of the correlated image Euler numbers. Chan-Vese active contours segmentation was then used and the number of iterations for segmentation was fixed to 4000. The terminating criterion for segmentation does not depend on the gradient of the image.

### Preprocessing

Preprocessing involves denoising of the image. Wavelet-based denoising is a well-known method in the field of digital image processing. Mean, Gaussian, anisotropic diffusion, Fourier transformation and the SURE orthonormal wavelet filters [21] were evaluated in this study for denoising of the images. Finally, orthonormal wavelet denoising of the image was used for this study as it provided best denoising effect with respect to visual inspection. The denoising process is parameterized as the sum of elementary non-linear processes with unknown weights. The estimate of mean square error (MSE) between denoised and clean image is minimized using Stein's unbiased risk estimate (SURE), a statistically unbiased estimate that depends only on the noisy image. The existence of this as a priori estimate avoids the requirement of statistical model for wavelet coefficients.

### Feature extraction

Textural (GLCM and Gabor) as well as other features, like Gist, HOG and PHOG were extracted from the digital CXRs. Details of the feature extraction methods used in this study are given below:

### GLCM textural features

Textural features are calculated based on the statistical distribution of combinations of pixel intensities at specific positions relative to each other. Based on the number of pixels in each combination, statistics are categorized into first-, second- and higher-order statistics. The GLCM method extracts second-order statistics textural features. GLCM is a matrix with rows and columns equal to the number of grey levels. The matrix element G(i,j | dx,dy) is the relative frequency with which two pixels occur within a given neighbourhood, where i and j are pixel intensities separated by distance (dx,dy). Various textural features were extracted from this matrix (for details refer to Table S1). A two pixel offset (2,0 and 0,2) was used in this study.

### Gabor features

Gabor filters have been found to be very effective in texture representation and discrimination [15], [22], [23]. These filters with different frequencies and orientations are used for extracting textural features from an image. A 2-D Gabor function g(x,y) and its Fourier transform G(u,v) are calculated using Eq. (1) and Eq. (2) respectively:



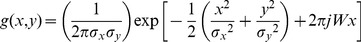
(Eq. (1))


Where, j  = 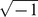, and W is the frequency of the Gabor function



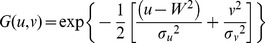
(Eq. (2))


Where 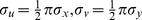


Impulse responses of Gabor filters are rotated and scaled versions of the above function. The Gabor filters can be considered as edge detectors with adjustable orientations and scales. A self similar filter dictionary is obtained by the association of rotation parameter θ and scale factor α with the Gabor function g(x,y). Scales and orientations of the Gabor wavelets are represented by M and N respectively.



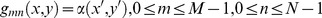
(Eq. (3))

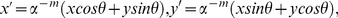
where 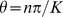 and K is the total number of orientations.

Gabor wavelet transform for a given image I(x,y) is defined as in Eq. (4):



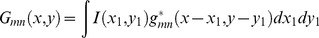
(Eq. (4))


Where, * represents complex conjugate.

Spatial homogeneity of local textural regions is assumed. µ_mn_ and σ_mn_ are the mean and standard deviation of the magnitude of Gabor wavelet transform coefficients respectively, and are used to represent regions for classification and retrieval of images.



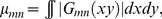
and



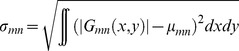
(Eq. (5))


Gabor features are created using µ_mn_ and σ_mn_ as feature components. Number of scales and orientations was set to six and ten respectively for creating feature vectors in this study. Frequency range was set between 0.01 and 0.4.

### Gist features

These are global image features and they assist in characterizing various important statistics of a scene. These features are computed by convoluting the filter with an image at different scales and orientations. Thus, high and low frequency repetitive gradient directions of an image can be measured. The scores for filter convolution at each orientation and scale are used as Gist features for an image. These features are currently being used for scene classification [14], [24]–[26]. The first step of Gist feature extraction is filtering of input image into a number of low level visual feature channels, like intensity, colour, orientation, motion and flicker at multiple spatial scales. Center-surround operations within each sub-channel i are performed between filter output maps, O_i_(s), at different scales s. This yields feature maps M_i_(c, s), given a “center” scale c and “surround” scale s. Across scale difference (Θ) between two feature maps is computed by interpolation to the center scale and pointwise absolute difference.

For color and intensity channels, feature maps are computed usingEq. (6):



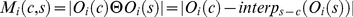
(Eq. (6))


Feature maps are used to detect conspicuous regions in each channel and are merged linearly to yield a saliency map. Information from the orientation channel is incorporated by employing Gabor filters to the grayscale image (Eq. (7)) at four spatial center scales (c = 0, 1, 2, 3) and at four different angles (θ_i_ = 0^°^, 45^°^, 90^°^, 135^°^).

For orientation channels, feature maps are computed using Eq. (7):



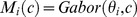
(Eq. (7))


After the computation of low-level center-surround features, each sub-channel extracts a gist vector from its corresponding feature map. Averaging operations are applied in a 4×4 grid of subregions over the map. Sixteen raw gist features (G_i_^k,l^(c, s)) are computed per feature map using Eq. (8):



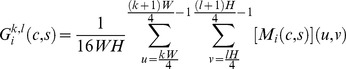
(Eq. (8))where W and H are width and height of the image respectively.

k and l are the indices in horizontal and vertical directions respectively.

12 orientations per scale and 8 blocks were used for this study.

### HOG features

This method was first introduced by Dalal and Trigg [16]. HOG breaks up a CXR image into small cells, computes the HOGs for each cell, normalizes HOGs using block pattern, and provides a descriptor for each cell [27]. These features are generally used for object detection in an image. The basic idea behind HOG features is that shape and appearance of local objects within an image can be described by the intensity gradients distribution. This method involves counting the occurrence of gradient orientation and thereby maintains photometric transformations and geometric invariance. The descriptor generation is comprised of four main steps: gradient computation, orientation binning, descriptor blocks generation, and block normalization. In gradient computation, the filtering of intensity or color data of the image is done with 1-D centred discrete derivative masks (D_x_ = [−1,0,1] and D_y_ = [−1,0,1]^T^) in horizontal and/or vertical directions. For a given image I, x and y derivatives are obtained using a convolution operation I_x_ = I * D_x_ and I_y_ = I * D_y._ Magnitude and orientation of the gradient is computed by the following equations Eq. (9) and Eq. (10) respectively:



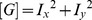
(Eq. (9))




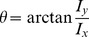
(Eq. (10))


Orientation binning involves the creation of a cell histogram. Weighted vote is cast by each pixel within a cell based on the gradient computation values. The histogram channels are uniformly spread over 0 to 360 or 0 to 180 degrees depending on signed or unsigned gradient respectively. The cells are then grouped together into spatially connected blocks to account for changes in contrast and illumination. These blocks usually overlap, and two main geometries R-HOG (rectangular) and C-HOG (circular) blocks exist. The blocks are normalised using one of the following normalization factors:

L1-norm: 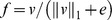)

L1-sqrt: 
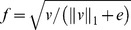


L2-norm: 
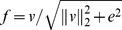


L2-hys: L2-norm equation with maximum value of ν limited to 0.2 followed by renormalization.

In this study, block size and number of bins were set to 6×6 and 9 respectively. R-HOG was used as it provided better classification accuracy as compared to C-HOG.

### PHOG features

These are spatial shape descriptors which describe an image by its spatial layout and local shape [17]. These descriptors are comprised of HOGs over each sub-region of an image at each pyramid resolution level. Local shapes in an image are captured by the distribution over edge orientations within a region while the spatial layout is captured by tiling an image into regions at multiple resolutions.

Initially, the HOG for entire image is computed. The bin size for HOG is fixed to N bins. The CXR is divided into a sequence of increasingly finer spatial grids by repetitively doubling the number of divisions along each axis. The number of points in each grid cell is stored. Since the number of points in a cell at one level is equal to the sum of points contained in the four cells it is divided into at the next level, this is a pyramid representation. HOG is computed for each of these levels. The bin count for the histogram representing a level is the cell count at that level of resolution. This process is repeated until a depth L. During pyramid formation, 2^L^ cells are present along each dimension for the grid at level L. PHOG features obtained for an image is a weighted combination of the above HOG features. The summation of calculated PHOG values is then normalized to unity to ascertain that texture rich images are not weighted more strongly than others. In this study, the number of bins on histogram and number of pyramid levels at 360 degrees were set to 8 and 3 respectively.

### Feature selection

Feature selection was performed using chi-square based method. Although several other feature selection methods such as correlation based feature selection, info gain and kernel principal component analysis were evaluated, but the performance of chi-square based feature selection method using WEKA software [28] was found to be the best with respect to classification results. This method uses chi-square statistics to discretize features repeatedly until some inconsistency is found in the data. Feature ranking was also done by this method and the best features were used for classification of a CXR as TB or non-TB.

### Classification

**Figure 5:**
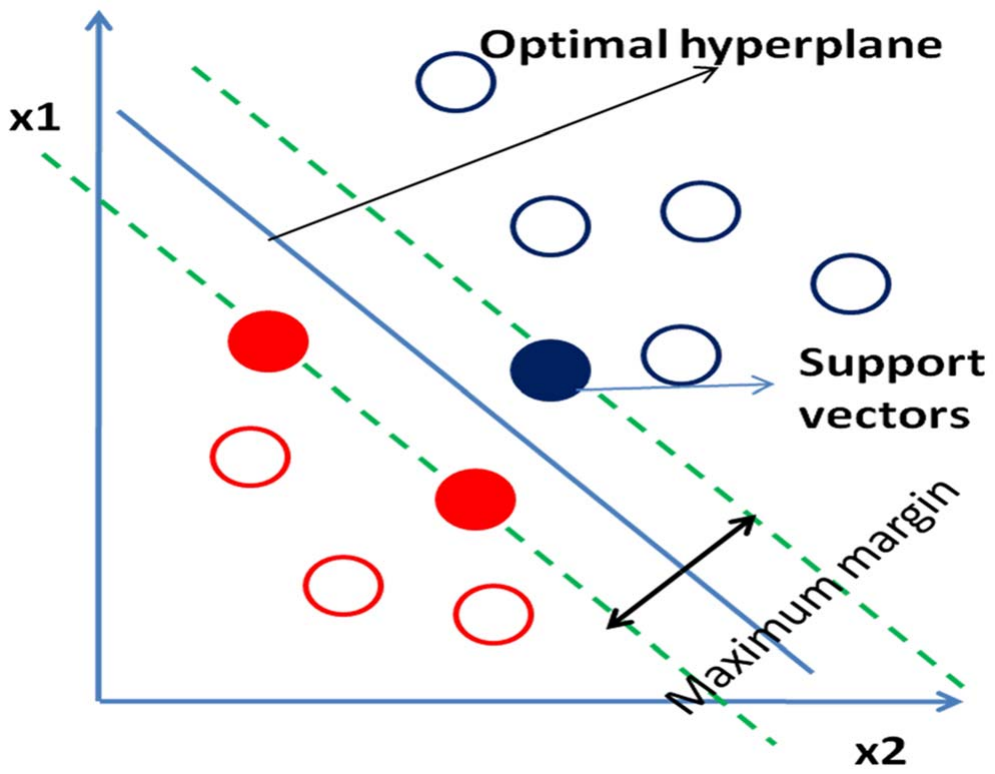
Hyperplane (blue line) representation in SVM. Red and blue circles represent data points from two different classes. Solid filled circles denote support vectors.



Support vector machines (SVMs) [29], a machine learning algorithm, is commonly used for classification and regression. The objective of SVM is to find the hyperplane which maximizes distance between data points from two classes. Training data points closest to the hyperplane are called support vectors (Figure 5). The hyperplane can be defined as:

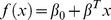
where β, β_0_ and x are the weight vector, bias and support vectors respectively.

The SVM is a non-probabilistic binary linear classifier, but it can also perform a non-linear classification using the kernel trick by mapping the data points into high-dimensional feature spaces. Some common kernel methods are polynomial homogeneous, polynomial inhomogeneous, Gaussian radial basis function and hyperbolic tangent. Gaussian radial basis function was used in the study. LIBSVM [30], a supervised machine learning software, was used for labelling (classification) of a given CXR as TB or non-TB.

## Results and Discussion

Current CAD methods use local GLCM textural features extracted from segmented CXRs for the classification of CXRs. Segmentation is an important step for the optimal extraction of features. Implementation of CAD method into X-ray machines for the automatic prediction requires features to be extracted from automatically segmented or whole CXR images. The ground truth that we were striving to reproduce by this CAD method for the two datasets was the judgement or decision of radiologists who read the CXRs.

### Performance of features extracted from automatically segmented CXRs

The performance of the GLCM textural features was quite low for the CXR dataset DA (Table 1). Prediction accuracy of only 65.4% was obtained for GLCM textural features extracted from automatically segmented CXRs, while 80.8% was obtained for manually segmented CXRs from dataset DA. The poor performance of the features extracted from automatic segmented CXRs may be attributed to severe intensity distortions [1], variability in lungs shape, and complex appearances of CXRs due to overlapping of anatomical structures.

**Table 1:** Performance of classification of CXRs dataset, DA using various feature extraction methods.

Feature*	Accuracy	Sensitivity	Specificity	Precision	F-score	GMean	AUC	CI#	SE#
Gabor^1^	0.865	0.808	0.923	0.913	0.857	0.864	0.888	0.769–0.958	0.0482
Gab^2^	0.865	0.885	0.846	0.852	0.868	0.865	0.919	0.809–0.976	0.0392
Gab^3^	0.712	0.654	0.769	0.739	0.694	0.709	0.771	0.633–0.876	0.0668
Gist^1^	0.942	0.961	0.923	0.925	0.943	0.942	0.957	0.861–0.994	0.0301
Gist^2^	0.885	0.846	0.923	0.917	0.880	0.884	0.905	0.792–0.969	0.0444
Gist^3^	0.827	0.808	0.846	0.840	0.824	0.827	0.868	0.746–0.946	0.0497
HOG^1^	0.865	0.846	0.885	0.880	0.863	0.865	0.904	0.790–0.968	0.0456
HOG^2^	0.846	0.769	0.923	0.909	0.833	0.843	0.880	0.760–0.954	0.0483
HOG^3^	0.865	0.846	0.885	0.880	0.863	0.865	0.907	0.793–0.970	0.0440
PHOG^1^	0.923	0.885	0.962	0.958	0.920	0.922	0.956	0.859–0.993	0.0294
PHOG^2^	0.827	0.769	0.885	0.870	0.816	0.825	0.859	0.735–0.940	0.0520
PHOG^3^	0.808	0.846	0.769	0.786	0.815	0.807	0.828	0.698–0.919	0.0577
GLCM^2^	0.808	0.769	0.846	0.833	0.800	0.807	0.859	0.735–0.940	0.0510
GLCM^3^	0.654	0.769	0.538	0.625	0.690	0.644	0.623	0.478–0.753	0.0803

*Features extracted from: ^1^ indicates whole CXR, ^2^ indicates manually segmented CXR and ^3^ indicates automatic segmented CXR.

# CI refers to confidence interval at 95% at P-value <0.0001 whereas SE refers to standard error for AUC.

### Performance of features extracted from whole-CXR and manually segmented CXRs

**Figure 6:**
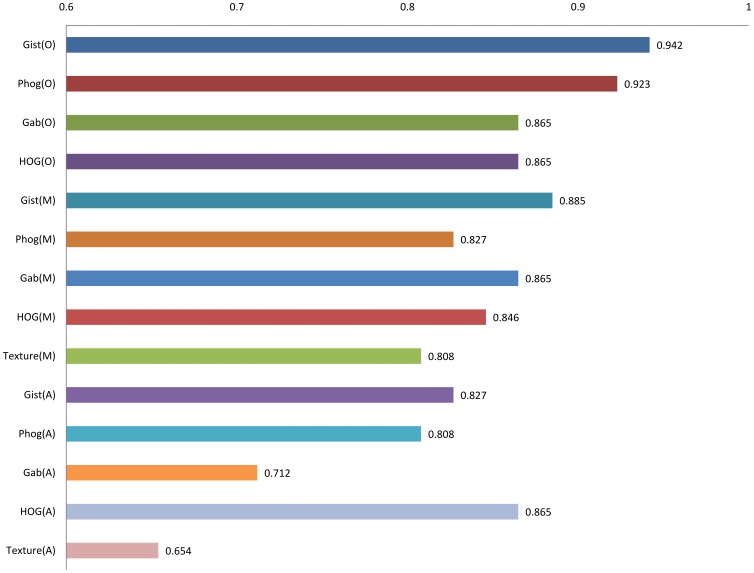
Comparison of performance for various features from whole, manually segmented, and automatically segmented CXRs.

Since low prediction accuracy was obtained using GLCM method, therefore, features extracted from the whole image, like Gabor, Gist, HOG, and PHOG were evaluated for their ability to discriminate between the TB and non-TB CXRs. The parameters were fine-tuned, so that the features relevant to TB discrimination were extracted. The Gabor, Gist, HOG, and PHOG features extracted from the whole CXR provided a prediction accuracy of 86.5%, 94.2%, 86.5%, and 92.3% respectively for dataset DA, and 92.0%, 86.0%, 86.0%, and 90% respectively for dataset DB (Table 1 and 2). The Gist features provided the maximum sensitivity of 0.961 for the dataset DA. This outcome is significant because sensitivity is considered as major criterion when measuring the effectiveness of a classification model for the detection of diseases. Although Gabor features provided comparable prediction accuracy to PHOG for dataset DB, but the latter provided significantly better 5-fold cross-validation accuracy of 86.6% in comparison to the former's 81.5%. Also, Gabor feature extraction is a computationally intensive process than PHOG method. For 5-fold cross-validation, the dataset were divided into five equal parts. One part was used for testing while the other four parts were used for the training of SVM. The procedure was repeated five times. The performance of features extracted from manually segmented images was comparable or lower than to those extracted from whole CXR image (Table 1). This may be attributed to the better spatial and contextual information provided by the whole CXR in comparison to the segmented one. Comparable performance between the whole and manually lung segmented CXRs indicated that these features (Gist, Gabor, HOG, and PHOG) were able to capture discriminative features without the need for segmentation of lungs (Table 1). Comparison of performance for various features is provided in the form bar graph in Figure 6.

**Table 2:** Performance of classification of CXRs dataset, DB using various feature extraction methods.

Feature*	Accuracy	Sensitivity	Specificity	Precision	F-score	GMean	AUC	CI#	SE#
Gab^1^	0.920	0.920	0.920	0.920	0.920	0.920	0.936	0.829–0.986	0.0377
Gist^1^	0.860	0.880	0.840	0.846	0.863	0.860	0.893	0.773–0.962	0.0520
HOG^1^	0.860	0.800	0.920	0.909	0.851	0.858	0.909	0.793–0.972	0.0434
PHOG^1^	0.900	0.920	0.880	0.885	0.902	0.900	0.918	0.806–0.977	0.0404

*Features extracted from: ^1^ indicates whole CXR, ^2^ indicates manually segmented CXR and ^3^ indicates automatic segmented CXR.

# CI refers to confidence interval at 95% at P-value <0.0001 whereas SE refers to standard error for AUC.

### Validation

The performance of various feature extraction methods used in this study is provided in Table 1 and 2. To validate the proposed methodology, five-fold cross validation and ROC curve analysis were done. PHOG features provided five-fold cross-validation accuracies of 83.2% and 86.6% for the datasets DA and DB respectively while 84.4% and 86.6% were obtained for the same datasets using Gist features.

**Figure 7:**
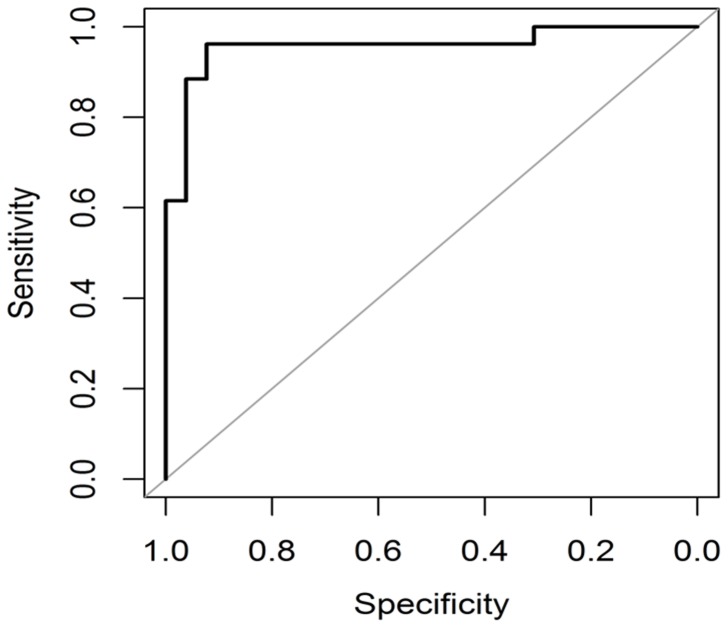
ROC plot for classification between TB and non-TB CXRs (DA) using Gist features.

**Figure 8:**
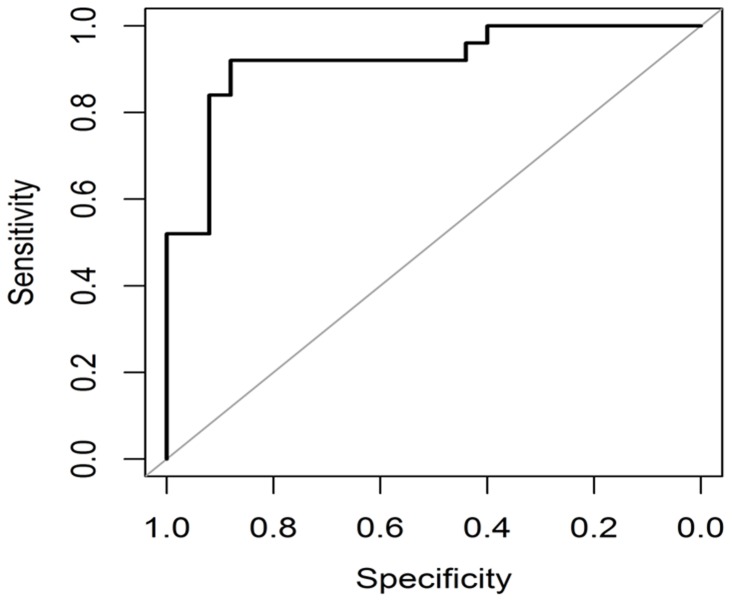
ROC plot for classification between TB and non-TB CXRs (DA) using PHOG features.

The AUC is a plot between true positive and false positive rates and it determines how well a model can distinguish between the TB and non-TB CXRs [31], [32]. Figure 7 and 8 show high value of AUC for both Gist and PHOG features indicating a good discrimination between the TB and non-TB CXRs [33]. Every point on the ROC curve represents a specificity and sensitivity pair corresponding to a certain decision threshold. Higher is the overall accuracy of classification; closer is the ROC curve to the upper left corner. When a classifier cannot discriminate between the two classes or groups, the AUC would be equal to 0.5. When there is a perfect discrimination, the AUC would be equal to 1. The 95% confidence interval (CI) is the interval in which the true AUC lies with 95% confidence. P-value is the probability that the observed AUC is found when actually the true AUC is 0.5. If P is smaller than 0.05, it can be concluded that the AUC is significantly different from 0.5 and the classifier has the ability to discriminate between the two classes. High AUC values were obtained (Table 1 and 2) at 95% confidence interval for our methods at P-level (AUC = 0.5) smaller than 0.0001. Standard error (SE) of AUC was also calculated using the method of Delong *et al*. [34] and SE values were found to be quite low (Table 1 and 2). As inferred from the results of this study, the Gist and PHOG features were quite robust and provided better results without segmentation.

### TB-Xpredict

**Figure 9:**
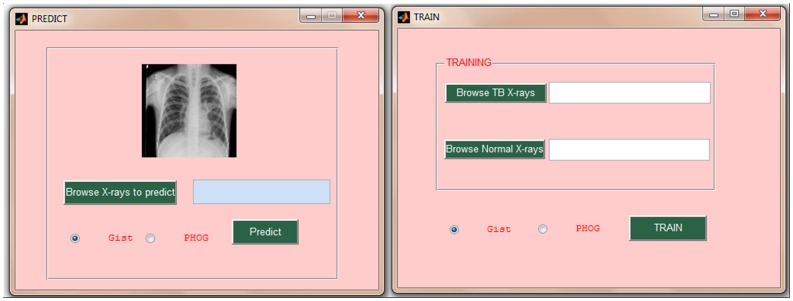
GUI of Prediction and Training Modules in Matlab toolbox, TB-Xpredict.

**Figure 10:**
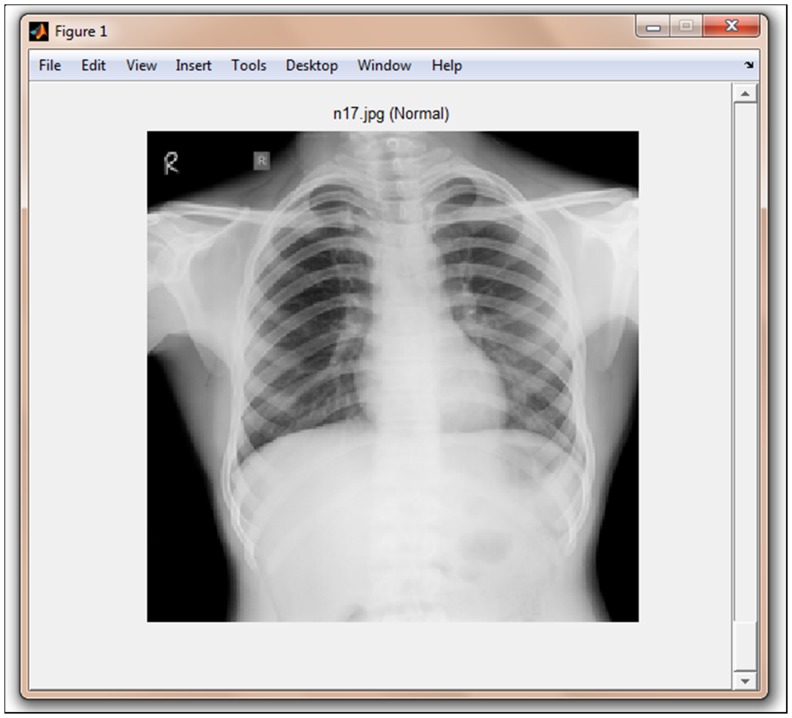
GUI of sample output provided by Matlab toolbox, TB-Xpredict.

Gist and PHOG features were implemented to develop TB-Xpredict, a MATLAB toolbox available at http://sourceforge.net/projects/tbxpredict/. It has a user-friendly graphical user interface (GUI), which provides both training and prediction modules (Figure 9 and 10). The former enables the user to develop a model trained on his/her own CXRs (TB and non-TB CXRs) while the latter can be used for the classification of digital CXR(s). The user can independently upload CXRs into the prediction module to classify them using already trained model provided with the software. Training and prediction modules of the toolbox are available for Gist and PHOG features separately with the use of Gist as default. The toolbox automatically identifies the bit size and file format (dicom or jpeg) of the CXR images.

Since TB-Xpredict does not require prior segmentation of digital CXR images, this toolbox can easily be incorporated into existing X-ray machines for the automated classification of CXR as TB or non-TB. X-ray machines with such modules can effectively be used for the mass screening of TB in high-burden areas. GUI of TB-Xpredict enables user to train and classify CXRs without the need of expertise in image processing. User only needs to upload positive and negative CXRs separately, and the module trains the model in an automatic manner. The newly developed model can be used for the prediction of CXRs using prediction module. Since illumination and other parameters do not differ significantly in test data with respect to training model, automated prediction methods which use CXRs from the same X-ray machine for training as well as classification generally may lead to better prediction accuracy. Furthermore, such model may become more efficient as the number and diversity of training data increases over time.

## Conclusions

CAD techniques provide second opinion to the clinicians for their findings, so its implementation is expected to improve their performance in the diagnosis of TB. Features extracted by methods such as Gabor, Gist, HOG, and PHOG enabled SVM to efficiently discriminate between the TB and non-TB whole CXR images. Validations of results using five-fold cross-validation and independent datasets have shown that the performance of these features is significantly higher than that of textural features (Table 1 and 2). CAD techniques proposed earlier were mainly based on the segmentation of CXRs followed by the extraction of GLCM textural features. The major limitation with these methods is that they cannot be incorporated into existing X-ray machines for automated detection of TB due to their dependency on manual or semi-automated segmentation. Although GLCM textural features extracted from the automatically segmented CXRs can be used for automated detection, however, the performance of this method is exceedingly poor (Table 1). Since the features (Gabor, Gist, HOG, and PHOG) extracted from the whole CXR do not require ROI identification using segmentation, these features can easily be implemented to existing digital X-ray machines for the automated TB detection. As Gist and PHOG features provided the best discrimination between the non-TB and TB for both 8-bit and 14-bit CXRs, these features were used to develop a MATLAB toolbox, TB-Xpredict, which can effortlessly train as well as predict CXRs as TB or non-TB. TB-Xpredict has a very simple GUI for automated training and classification of CXRs, and requires only basic knowledge of computer to operate. It also gives users the option to choose between Gist and PHOG for feature extraction. One of the limitations of the toolbox is that since TB has similar radiological patterns to cancer and some interstitial lung diseases (ILDs), it may wrongly classify CXRs with these diseases as TB CXRs. This toolbox will assist in better diagnosis as well as efficient mass screening of TB in high burden areas.

## Supporting Information

Table S1 **Various grey-level co-occurrence matrix (GLCM) textural features used for the classification.**
(DOCX)
