# Supplementary material for: Role of Gist and PHOG Features in Computer-Aided Diagnosis of Tuberculosis without Segmentation
Source: PLoS One. 2014 Nov 12;9(11):e112980. doi: 10.1371/journal.pone.0112980 (PMC4229306; doi:10.1371/journal.pone.0112980)
Supplement: Table S1 — Various grey-level co-occurrence matrix (GLCM) textural features used for the classification. (DOCX) [file pone.0112980.s001.docx]

| S. No. | Textural Feature | Function |
| --- | --- | --- |
| 1. | Contrast | $\sum_{n=0}^{n_{g}-1} n^{2}\left\{\sum_{i=1}^{n_{g}} \sum_{j=1}^{n_{g}} p\left(i,j\right)\right\},\left\vert i-j\right\vert=n$ |
| 2. | Correlation | $\frac{\sum_{i} \sum_{j} \left(i,j\right)p\left(i,j\right)-\mu_{x}\mu_{y}}{\sigma_{x}\sigma_{y}}$ |
| 3. | Sum of Squares | $\sum_{i} \sum_{j} \left(i-\mu\right)^{2}p\left(i,j\right)$ |
| 4. | Inverse Difference moment | $\sum_{i} \sum_{j} \frac{1}{1+\left(i-j\right)^{2}}p\left(i,j\right)$ |
| 5. | Sum Average | $\sum_{i=2}^{2n_{g}} ip_{x+y}\left(i\right)$ |
| 6. | Sum Variance | $\sum_{i=2}^{2n_{g}} \left(i-f_{s}\right)^{2}p_{x+y}\left(i\right)$ |
| 7. | Sum Entropy | $-\sum_{i=2}^{2n_{g}} p_{x+y}\left(i\right)\log\left\{p_{x+y}\left(i\right)\right\}$ |
| 8. | Entropy | $-\sum_{i} \sum_{j} p\left(i,j\right)\log\left(p\left(i,j\right)\right)$ |
| 9. | Difference Variance | $\sum_{i=0}^{n_{g}-1} i^{2}p_{x-y}\left(i\right)$ |
| 10. | Difference Entropy | $-\sum_{i=0}^{n_{g}-1} p_{x-y}\left(i\right)\log\left\{p_{x-y}\left(i\right)\right\}$ |
| 11. | Info. Measure of Correlation 1 | $\frac{HXY-HXY1}{\max\left\{HX,HY\right\}}$ |
| 12. | Info. Measure of Correlation 2 | $\sqrt{\left(1-\exp\left[-2\left(HXY2-HXY\right)\right]\right)}$ |
| 13. | Max. Correlation Coefficient | Square root of the second largest eigenvalue of Q, where Q(i,j)= $\sum_{k} \frac{p\left(i,k\right)p\left(j,k\right)}{p_{x}\left(i\right)p_{y}\left(k\right)}$ |
| 14. | Inertia | $\begin{matrix} \\ \sum_{i=0}^{n_{g}-1} \sum_{j=0}^{n_{g}-1} \left\{i-j^{2}\right\}*p\left(i,j\right) \end{matrix}$ |
| 15. | Cluster Shade | $\sum_{i=0}^{n_{g}-1} \sum_{j=0}^{n_{g}-1} \left\{i+j-\mu_{x}-\mu_{y}\right\}^{3}*p\left(i,j\right)$ |
| 16. | Cluster Prominence | $\sum_{i=0}^{n_{g}-1} \sum_{j=0}^{n_{g}-1} \left\{i+j-\mu_{x}-\mu_{y}\right\}^{4}*p\left(i,j\right)$ |

Where, n_g_ refers to the number of grey levels in the image. i and j refer to pixel values. µ_x_ &µ_y_ and σ_x_ & σ_y_ are the means and standard deviations of partial probability density functions p_x_ and p_y_. x and y are the coordinates of an pixel in the matrix and p_x+y_(i) is the combination probability of x and y. HXY=$-\sum_{i} \sum_{j} p\left(i,j\right)\log\left(p\left(i,j\right)\right)$. HX and HY are the entropies of p_x_ and p_y_, HXY1=$-\sum_{i} \sum_{j} p\left(i,j\right)\log\left\{p_{x}\left(i\right)p_{y}\left(j\right)\right\}$ and HXY2=$-\sum_{i} \sum_{j} p_{x}\left(i\right)p_{y}\left(j\right)\log\left\{p_{x}\left(i\right)p_{y}\left(j\right)\right\}$.
